# Role of peripheral blood minimum residual disease at day 8 of induction therapy in high-risk pediatric patients with acute lymphocytic leukemia

**DOI:** 10.1038/srep31179

**Published:** 2016-08-16

**Authors:** Thais Ditolvo da Costa Salina, Yvelise Antunes Ferreira, Eliana Brasil Alves, Cristina Motta Ferreira, Erich Vinícius De Paula, Marcelo Távora Mira, Leny da Mota Passos

**Affiliations:** 1 Hematology and Hemotherapy Foundation of Amazonas (HEMOAM), Manaus, AM, Brazil; 2 Federal University of Amazonas (UFAM), Manaus, Amazonas, Brazil; 3 Hematology and Hemotherapy Center, University of Campinas, Campinas, São Paulo, Brazil; 4 Pontifícia Universidade Católica do Paraná, Graduate Program in Health Sciences, Curitiba, Parana, Brazil

## Abstract

Risk stratification and treatment intensification, based on minimal residual disease (MRD) mensurement, changed the prognosis of pediatric patients with acute lymphocytic leukemia (ALL). The main aim of this study was to investigate whether peripheral blood (PB) MRD measurement at day 8 (D8) could predict the risk stratification category determined by bone marrow (BM) MRD at day 15 (D15). The study was performed prospectively, in a cohort of 40 children with B-lineage ALL, adopting the protocol of the Brazilian Cooperative Group of the Treatment Childhood Leukemia (GBTLI-2009). MRD was detected by flow cytometry (FC) using a simplifed panel that can reliably identify MRD at early phases of induction therapy. Upon diagnosis, the proportion of low and high-risk patients, was 24:16 (60%:40%). The main result of our study demonstrated the potential of D8 MRD in anticipating of week the risk stratification of high-risk patients as determined by D15 BM MRD. In these patients D8 MRD level of 1% was able to segregate high risk fast responders from high risk slow responders (p = 0.0097). This result could represent an opportunity for early treatment intensification, as already performed in some protocols.

The recent increase in survival rates observed in acute lymphocytic leukemia (ALL) is due to advances in diagnosis, identification of critical prognostic factors and definition of treatment according to risk groups^1^,^2^,^3^. At diagnosis, the most important parameters for risk stratification, with minor differences across different therapy protocols, are age, white blood cell count, immunophenotype, presence of lymphoblasts in the central nervous system and molecular analysis of chromosomal abnormalities^4^,^5^. In addition, early response to induction chemotherapy is also a critical prognostic factor.

Historically, therapeutic response was assessed mostly by morphological count of blasts in peripheral blood (PB) and bone marrow (BM). However, due to the fact that patients in morphologic remission may present morphologically undetectable amounts of residual leukemic cells, referred as “Minimal Residual Disease” (MRD), different strategies using flow cytometric or molecular techniques have been used to detect these cells in the last decades^5^,^6^,^7^. MRD measurement is actually superior to other traditional markers of disease and based in its result, is possible to optimize chemotherapy, minimizing toxicity and decreasing risk of relapse^3^,^4^,^5^,^8^,^9^.

Current ALL protocols associate morphological analysis with MRD measurement to evaluate treatment response on both early and late time-points, providing more timely and accurate prognostic information of treatment outcome^2^,^4^,^10^. However, on day 8 (D8), the earliest time point of assessment of therapeutic response, most protocols use morphological analysis of PB for risk stratification, with good and poor responses defined as PB leukemic blast counts below and above 1,000/mm^3^, respectively^11^. In order to refine this D8 evaluation, the Children’s Oncology Group (COG) protocol adopted MRD analysis to guide treatment in this earliest time point, with positive outcomes on treatment response^1^. However, MRD evaluation at D8 has not been widely adopted, and several protocols still rely on morphological analysis. Here, we present the results of an independent study to evaluate the impact of D8 MRD measurement on risk stratification in a prospective cohort of 40 patients with ALL.

## Patients and Methods

### Study design and definitions

From January 2014 to January 2015, all children newly diagnosed with B-lineage ALL and admitted for treatment at our institution, which is the only institution that provides care for children with leukemia in the State of Amazonas in northern Brazil, were recruited for this study. T-lineage ALL and AML were excluded by immunophenotypical criteria based in a broad panel of monoclonal antibodies at diagnosis. The characterization of genetic abnormalities in bone marrow samples was performed by conventional cytogenetics at diagnosis. As per protocol definitions, alterations indicating the presence t(9;22) which is associated with BCR-ABL1, or t(4;11), which is associated with MLL rearranged were defined as high risk cytogenetic abnormalities.

After admission, parents/legal guardians were invited to participate and provided written informed consent. The study was approved by the Committee of Ethics in Research of the Hematology and Hemotherapy Foundation of Amazonas (HEMOAM) and performed in accordance with the Declaration of Helsinki. Diagnosis was based on morphological and immunophenotypical criteria and patients were treated according to the guidelines of the Brazilian Cooperative Group for the Treatment of Childhood Leukemia (GBTLI-2009) (supplementary Figures 1 and 2). Patients were initially stratified as low or high risk at diagnosis according to clinical and laboratory criteria shown in Table 1.

**Figure 1:**
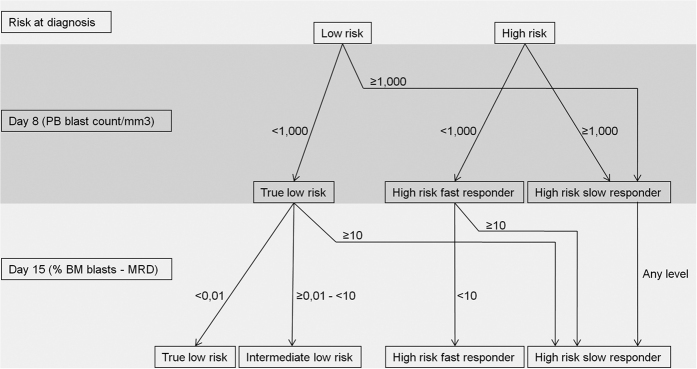
Restratification during induction. Risk restratification criteria at days 8 and 15 of induction therapy, according to Brazilian Cooperative Group for the Treatment of Childhood Leukemia 2009 (GBTLI 2009).

Then, during induction, patients were restratified according to response to chemotherapy. As shown in Fig. 1, low risk patients at diagnosis were restratified as true low risk, intermediate low risk or high risk slow responders. On the other hand, high risk patients at diagnosis were restratified as high risk fast responders or high risk slow responders.

### Laboratory analysis and interpretation

During induction, PB samples were obtained at D8 and BM samples at day 15 (D15) to measure MRD by flow cytometry (FC) according to previously published criteria^2^,^3^,^10^. Briefly, MRD measurement was performed using 4-colour flow cytometry analyzer (FacsCalibur, Becton Dickinson, San Jose, CA, USA) using simplified FC panel of monoclonal antibodies that can be used into non-regenerative phases of treatment^3^ (Table 2). This simplified flow cytometric assay is based on expression of CD19, CD10, and CD34 antigens, that can reliably identify MRD at early phases of remission induction therapy. According to Coustan-Smith *et al*., normal lymphoid progenitors (CD19, CD10, and/or CD34) are exquisitely sensitive to corticosteroids and other antileukemic drugs. Therefore, cells with this phenotype detected early in treatment of patients with B-lineage ALL should be leukemic rather than normal cell^3^.

The number of residual leukemic cells was calculated as the percentage of blasts present within the total of nucleated cells counted. After gating on CD19 cells with lymphoid morphology (as defined by their forward and side light-scattering properties), we determined the percentages of CD19 cells expressing CD10 and/or CD34. Sensitivity, determined upon dilutions of leukemic blasts in normal BM or PB, was 0,01%. Morphology was analyzed at the same time points by an investigator blinded to MRD results.

Based on BM MRD at D15, patients were assigned to different risk stratification groups, according to GBTLI-2009 criteria, presented above^12^. Next, the association between PB MRD at D8 and risk stratification at D15 was examined.

### Statistical analysis

Descriptive statistics were used to report demographical and clinical data. Data are expressed as median, ranges, means and SD. Differences in continuous variables were analyzed using the Mann-Whitney test. Categorical variables were compared using the Fisher’s exact test. A *p value* less than or equal to 0.05 was considered statistically significant. All statistical analyses were performed using the GraphPad Prism Software (GraphPad Prism Software Inc. San Diego, California, USA).

## Results

Our study population, which represents all children treated for ALL during the study period in the State of Amazonas, Brazil, consisted of 40 patients (23 males; 17 females) with a median age of 3.5 years (range 1–17). Additional social-demographic, clinical and laboratorial characteristics of the patients are shown in Tables 3 and 4.

**Figure 2:**
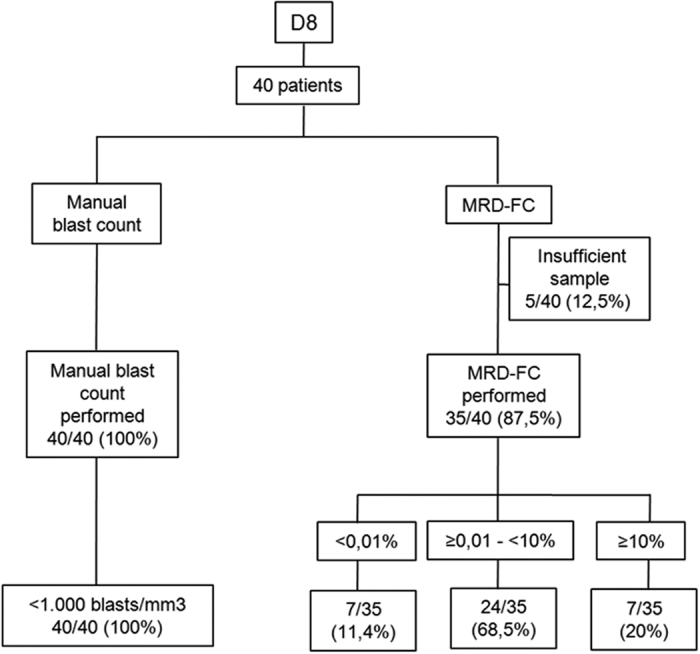
Comparison of manual blast count and MRD levels at day 8. Flowchart of the comparative analysis between manual blast count and PB MRD detected by flow cytometry (MRD-FC) at day 8.

Upon diagnosis, the proportion of low and high-risk patients, based on GBTL-2009 criteria was 24:16 (60%:40%). A flowchart of enrolled patients is shown in Fig. 2. At D8, PB MRD data was available for 35 out of 40 patients, with the five remaining patients evaluated only by morphology at this time-point. Although at D8 manual blast count all patients presented less than 1,000 blasts/mm^3^, PB MRD levels revealed a more heterogenous response in terms of MRD levels, with 68,5% of the patients presenting MRD between 0,01% and 10%; 20% presenting MRD ≥10%, and only 11,4% pesenting negative (MRD ≤0,01%) (Fig. 2).

**Figure 3:**
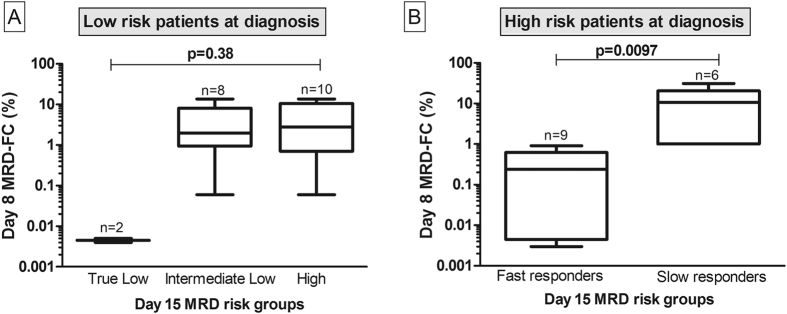
Comparison of Day 8 MRD-FC with Day 15 MRD risk groups. Patients stratified as low risk at diagnosis (3A) were reclassified as true low risk, intermediate low risk, or high risk according to MRD levels at day 15, based on the GBTLI-2009 protocol. Patients stratified as high risk at diagnosis (3B) were reclassified as fast or slow responders, according to MRD levels at day 15, based on the GBTLI-2009 protocol. Minimal residual disease measured by flow cytometry at day 8 (MRD-FC D8) for each of these subgroups is showed.

Finally, we assessed whether PB MRD measurement at D8 could predict the risk stratification category determined by BM MRD at D15, thereby anticipating this important information (Fig. 3). In Fig. 3A, PB MRD at D8 from low-risk patients (at diagnosis) is compared between the three risk stratification groups, as determined by levels of BM MRD at D15 (as previously detailed). Similar PB MRD levels at D8 were observed between patients from intermediate low risk and high risk. In contrast, the two patients assigned to the “true low risk” category by BM MRD at D15 were negative for PB MRD at D8 (Fig. 3A). Unfortunately, the low number of patients in this category precluded a formal statistical analysis. In Fig. 3B, PB MRD at D8 from high-risk patients (at diagnosis) is compared to fast and slow responders, according to D15 MRD (Fig. 1). Interestingly, a statistically significant difference of PB MRD levels could be detected at D8 (P = 0.0097). Of note, all high risk slow responders presented MRD level below 1% at D8.

## Discussion

Risk stratification and selected treatment intensification based on MRD detection have changed the prognosis of ALL patients^11^. However, at D8, most protocols still use morphological criteria to evaluate treatment response, leaving more sensitive techniques for later time-points. However, the feasibility and potential benefits of using MRD measurement as early as D8 have been demonstrated by the COG group in 2008. This group demonstrated that patients who had high levels of D8 PB MRD (>1%) had a poor outcome even if they cleared their BM of MRD by the end of induction, especially in high risk patients^1^. Here, we aimed to evaluate whether the anticipation of MRD measurement from D15 to D8, using PB samples, could add relevant information to the management of patients with ALL, and thereby validate the COG findings in an independent cohort treated with a different protocol.

Our study was performed prospectively, in a population of consecutive patients, from a single center responsible for the treatment of all patients with ALL in the State of Amazonas, Brazil, an area with approximately 4 million inhabitants. The study was conducted in a relatively short time span, and all patients adopted the same treatment guidelines. The main result of our study was the demonstration that D8 MRD measured in PB samples could anticipate in one week the risk stratification of high-risk patients with ALL that was later determined by BM MRD, at D15. This result could represent an opportunity for early treatment intensification, as already performed in some protocols.

The potential of D8 MRD measurement to refine risk stratification during early phases of ALL treatment was clearly evident for patients that were classified as high-risk according to GBTLI criteria at diagnosis. In these patients D8 MRD level of 1% was able to segregate high risk fast from slow responders as early as at D8 in our population. A similar finding was described in a study of childhood ALL in the UK, which identifed the same MRD-FC level at D8 to segregate high risk from low risk pacients^13^.

In our low-risk patients this finding could not be confirmed, maybe due to the limited number of patients in this group. However, the fact that none of the patients from the “true low-risk” group presented PB MRD at D8 suggests that this strategy could also be informative in this group, which should be confirmed in additional studies.

Our study highlights the potential of D8 MRD measured in PB samples for anticipating in one week the risk stratification of high-risk patients with ALL, as determined by BM MRD at D15. This result could represent an opportunity for early treatment intensification, as already performed in some protocols. However, due to the short follow-up and relatively limited number of patients we are not able to test the impact of these results in clinically-relevant outcomes such as event-free and overall survival. We are following this population currently and increasing the cohort to obtain this data. Validation in a larger cohort of patients is still required. Based on future results, it will be possible to evaluate if treatment intensification, based on D8 MRD, should be incorporated to the Brazilian protocol schedule.

“Furthermore, another limitation of our study is the limited genetic characterization of our population, which was based only on conventional cytogenetics. It has been shown that the identification of additional submicroscopic genetic alterations, and the use of more sensitive techniques could further improve the stratification of childhood ALL, an issue that is still under investigation in prospective studies^14^. However, several recent studies about childhood ALL still rely only on clinical, hematological and cytogenetic parameters for risk stratification^15^,^16^,^17^,^18^. In addition, limitations in the access to molecular biology techniques in some areas of Brazil (which is likely to be the case of a large proportion of treatment centers around the globe), also preclude the immediate adoption of some diagnostic tools in ongoing protocols. Therefore, while we acknowledge that it is not possible to rule out that the incorporation of more sensitive genetic markers could have changed our results, we believe that the well-established power of cytogenetics to define prognosis in childhood ALL^19^,^20^,^21^,^22^, and its widespread use in several recent reported clinical trials, makes this possibility less likely.

In conclusion, our results validate, in an independent population using a different protocol, that MRD measurement at D8 of induction therapy in children with ALL can anticipate the information obtained by MRD only at D15 for high-risk patients.

## Additional Information

**How to cite this article**: Salina, T. D. C. *et al*. Role of peripheral blood minimum residual disease at day 8 of induction therapy in high-risk pediatric patients with acute lymphocytic leukemia. *Sci. Rep.* **6**, 31179; doi: 10.1038/srep31179 (2016).

## Supplementary Material

Supplementary Information

## Figures and Tables

**Table 1:** Risk stratification criteria at diagnosis, according GBTLI 2009*.

Risk parameter	Low risk (LR)	High risk (HR)
Age, years	≥1 and <9	<1 or ≥9
White blood cell count	<50,000/mm^3^	≥50,000/mm^3^
Lymphoblasts in the central nervous system	Absent	Present
Cytogenetics	Absence of high-risk cytogenetic findings	Presence of high-risk cytogenetic findings t(9;22) BCR/ABL or t(4;11) MLL/AF4

^*^Brazilian Cooperative Group for the Treatment of Childhood Leukemia^12^; the presence of any high risk criteria is sufficient to classify patients in this category, while, the absence of all high-risk parameters is required to incorporate in low risk strata.

**Table 2:** Panel of monoclonal antibodies to identify minimal residual disease.

Test-tube	FITC	PE	PerCP	APC
1	CD45 (20 uL)	CD10 (15 uL)	CD34 (15 uL)	CD19 (4 uL)
2	CD45 (20 uL)	IgG-1 (15 uL)	CD34 (15 uL)	CD19 (4 uL)
3	Syto-13^®^*	—	—	CD19 (4 uL)

^*^SYTO^®^ 13 Green Fluorescent Nucleic Acid Stain: molecular probe from Invitrogen used to assess cell viability. All antibodies were purchased from Biotech, Exbio.

**Table 3:** Social-demographic data of the study population.

Variables (n = 40)	n	%
Gender		
Female	17	42.5
Male	23	57.5
Age (median = 3,5 years)		
<9 years old	30	75.0
≥9 years old	10	25.0
Ethnicity		
Caucasian	17	42.5
Non-caucasian	23	57.5

n = simple absolute frequency.

**Table 4:** Clinical and laboratory characteristics of the study population.

Variables (n = 40)	n	%
Hepatomegaly	22	55.0
Esplenomegaly	20	50.0
Adenomegaly	17	42.5
Leucocyte count at diagnosis		
<50,000/mm^3^	32	80.0
≥ 50,000/mm^3^	8	20.0
Lymphoblasts in the central nervous system		
Positive	2	5.0
Negative	38	95.0
Immunophenotype		
ALL B Calla positive	40	100.0
Cytogenetics		
Metaphase absent	16	40.0
Metaphase present	24	60.0
Hyperploidia	5/24	20.8
t(9;22)	2/24	8.4
Normal	17/24	70.8

n = simple absolute frequency.
