# Supplementary material for: Role of peripheral blood minimum residual disease at day 8 of induction therapy in high-risk pediatric patients with acute lymphocytic leukemia
Source: Sci Rep. 2016 Aug 16;6:31179. doi: 10.1038/srep31179 (PMC4985643; doi:10.1038/srep31179)
Supplement: Supplementary Information [file srep31179-s1.doc]

**Role of peripheral blood minimum residual disease at day 8 of induction therapy in high-risk pediatric patients with acute lymphocytic leukemia**

Authors: Thais Ditolvo da Costa Salina^1,2^, Yvelise Antunes Ferreira^1^, Eliana Brasil Alves^1,2^, Cristina Motta Ferreira^1^, Erich Vinícius de Paula^1,3^, Marcelo Távora Mira^1,4^, Leny da Mota Passos^1^*

Affiliations:

^1^Hematology and Hemotherapy Foundation of Amazonas (HEMOAM), Manaus, AM, Brazil

^2^ Federal University of Amazonas (UFAM), Manaus, Amazonas, Brazil

^3^ Hematology and Hemotherapy Center, University of Campinas, Campinas, São Paulo, Brazil

^4^ Pontifícia Universidade Católica do Paraná, Graduate Program in Health Sciences, Curitiba, Parana, Brazil.

*Corresponding author

Hematology and Hemotherapy Foundation of Amazonas (HEMOAM)

Address: Constantino Nery Avenue, 4397. Chapada - Manaus/Amazonas/Brazil

CEP: 69043-600.

Phone: +55 92 3521 8740

Email: yveantunes@hotmail

**Supplementary material**


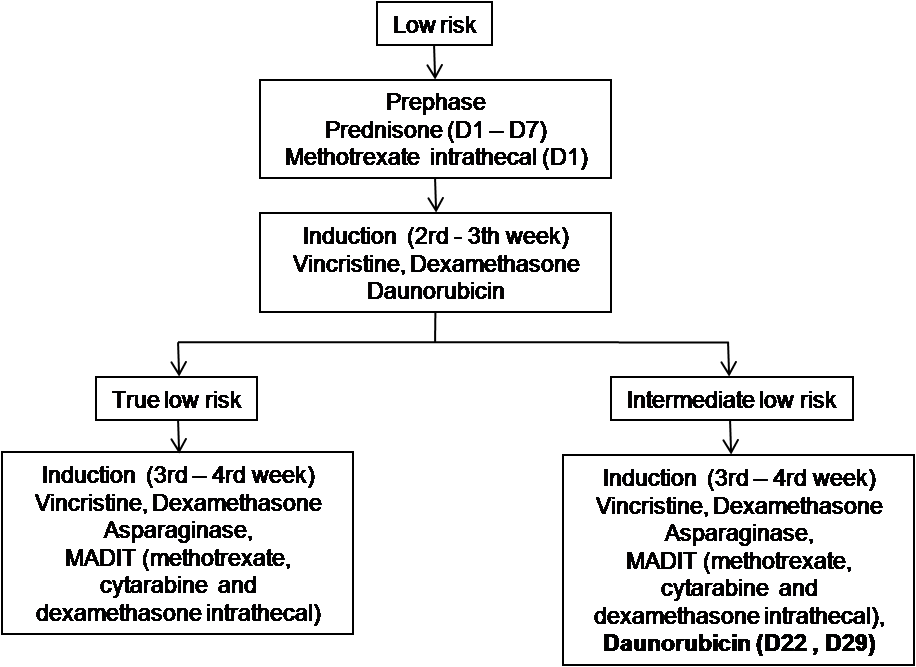


Figure 1. **Treatment scheme for low-risk group according to the Brazilian Cooperative Group of the Treatment Childhood Leukemia (GBTLI-2009)**


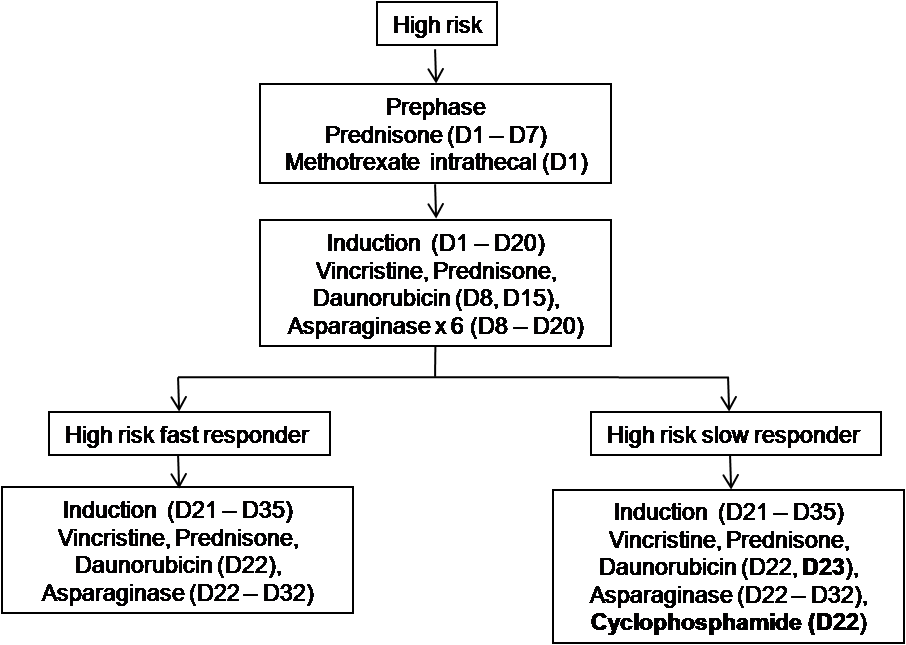


Figure 2. **Treatment scheme for high-risk group according to the Brazilian Cooperative Group of the Treatment Childhood Leukemia (GBTLI-2009).**
